# Associations between muscle-building exercise and concurrent e-cigarette, cigarette, and cannabis use among U.S. adolescents

**DOI:** 10.1371/journal.pone.0278903

**Published:** 2022-12-28

**Authors:** Kyle T. Ganson, Rachel F. Rodgers, Stuart B. Murray, Jason M. Nagata

**Affiliations:** 1 Factor-Inwentash Faculty of Social Work, University of Toronto, Toronto, Ontario, Canada; 2 Department of Applied Psychology, APPEAR, Northeastern University, Boston, Massachusetts, United States of America; 3 Department of Psychiatric Emergency & Acute Care, Lapeyronie Hospital, CHRU Montpellier, France; 4 Department of Psychiatry and the Behavioral Sciences, University of Southern California, Los Angeles, California, United States of America; 5 Department of Pediatrics, University of California, San Francisco, San Francisco, California, United States of America; Istanbul University Cerrahpasa Faculty of Medicine: Istanbul Universitesi-Cerrahpasa Cerrahpasa Tip Fakultesi, TURKEY

## Abstract

Physical activity and team sports may be protective of substance use among adolescents, although there is mixed evidence on whether muscle-building exercise is associated with patterns of e-cigarette use (i.e., vaping), cigarette use, and cannabis use. Therefore, this study aimed to determine the association between muscle-building exercise and patterns of concurrent substance use among U.S. adolescents. Cross-sectional data from the 2019 Youth Risk Behavior Survey (N = 8,474) were analyzed in 2022. Muscle-building exercise was assessed by number of days of the behavior in the past week and categorized based on level of engagement (none, low, medium, and high). Concurrent vaping, cigarette use, and cannabis use within the past 30 days were assessed using a combined, four-category variable (no use, any single use, any dual use, and triple use). Multinomial logistic regressions, with coefficients transformed to relative risk ratios (RRR), were conducted to estimate the associations between muscle-building exercise and concurrent substance use among the overall sample, and by sex, while adjusting for relevant sociodemographic variables. Among the overall sample, high engagement (6–7 days) in muscle-building exercise was associated with greater relative risk of any single use (RRR 1.36, 95% confidence interval [CI] 1.06–1.72), any dual use (RRR 1.46, 95% CI 1.10–2.94), and triple use (RRR 1.81, 95% CI 1.05–3.12). While muscle-building exercise was associated with greater relative risk of concurrent patterns of vaping, cigarette use, and cannabis use among adolescent males, there were no significant relationships found among adolescent females. Healthcare professionals should consider this association when treating adolescent males, particularly given the high prevalence of muscle-building exercise and substance use among this group. More research is needed to understand the experiences of adolescent males who report high engagement in muscle-building exercise and substance use to uncover mechanisms of association.

## Introduction

Use of e-cigarettes (i.e., vaping), combustible cigarettes (referred to as cigarettes hereafter), and cannabis within the past 30 days is relatively common among adolescents [1]. Indeed, many adolescents report engaging in varying patterns of concurrent use, including both dual and triple use of these substances [2]. This is cause for concern and potentially problematic as the use of these substances can lead to further illicit substance use and adverse health and social effects [3–7]. While recent research has reported on relevant risk and protective factors associated with varying patterns of vaping, cigarette use, and cannabis use among adolescents [2], less research has specifically investigated how various forms of physical activity be connected with substance use behaviors.

The limited prior research assessing the role of physical activity in relation to substance use among adolescents, including vaping, cigarette use, and cannabis use, is mixed. Some studies have indicated that physical activity and sports participation are primarily protective of cigarette and cannabis use among adolescents [8–12]. Conversely, however, several of these studies have shown that sports participation and physical activity were positively associated with vaping, as well as dual use [9,11,12]. The patterns of association found in these studies are likely explained in part by the types of sports engaged in, such high contact versus low contact sports, and high aerobic activities versus low aerobic physical activities.

Overall, this research is limited in that few studies have assessed the associations between muscle-building exercise specifically and multiple forms of vaping, cigarette use, and cannabis use (e.g., single use, and concurrent dual and triple use) among a nationally representative sample of adolescent boys and girls in the U.S. Investigating this association is warranted as muscle-building exercise is used for different purposes (i.e., the building of strength and muscle) than general physical activity that may be intended for overall health purposes (i.e., improving cardiovascular health) [13]. Thus, the cardiovascular effects of vaping, cigarette use, and cannabis use may be less important to adolescents who engage in exercise for muscle-building purposes. Muscle-building exercise is more common among males than females [14], particularly at higher levels of engagement (i.e., greater than 5 days per week) [15]. This is in part due to the male sociocultural body ideal emphasizing bulk muscularity and leanness [16]. However, engagement among females may be influenced by a shifting sociocultural body ideal transitioning away from emphasizing thinness to one that is fit and toned [17,18].

Limited prior research has documented differences in engagement in muscle-building exercise and related-behaviors, as well as vaping, cigarette use, and cannabis use, by sex [8,14,15,19]. Specifically, research has shown that male and female dual users of e-cigarettes and cigarettes are less likely to engage in muscle-building exercise [8]. Taken together this research underscores that muscle-building exercise may be associated with other problematic behaviors (i.e., patterns of vaping, cigarette use, and cannabis use). Additionally, given differing levels of engagement in muscle-building exercise and substance use behaviors between male and female adolescents [1,14,15], assessing the relationship between these behaviors by sex is needed.

The aims of this study were to determine whether muscle-building exercise is associated with patterns of concurrent use of vaping, cigarette use, and cannabis use overall and by sex among a nationally representative sample of U.S. adolescents. It was hypothesized that there would be positive associations between engagement in muscle-building exercise and patterns of concurrent use, particularly among males. That basis of this hypothesis is founded on problem behavior theory, which posits that engagement in one problem behavior increases the likelihood of engagement in other problem behaviors [20,21]. From this theoretical lens, it may be that muscle-building exercise, particularly high engagement, and concurrent substance use cluster as problem behaviors. Additionally, and particularly among males, engagement in muscle-building exercise and concurrent substance use may both be performative acts to display adherence to masculine norms (i.e., toughness, strength, dominance, risk taking) [22,23]. From this theoretical perspective, males may engage in attempts to increase their muscularity (via muscle-building exercise) and patterns of current use (i.e., risk-taking behaviors) to display their masculinity [22,24,25]. Findings from this study will add to the growing literature on the behavioral correlates of physical activity, specifically muscle-building exercise, among adolescents.

## Methods

Data from the 2019 National High School Youth Risk Behavior Survey (YRBS; N = 13,677) were analyzed for this study. This study only included participants who responded to the muscle-building exercise item (N = 8,474) with a total of 5,203 missing responses. The YRBS is conducted bi-annually by the Centers for Disease Control and Prevention (CDC) to monitor priority health risk behaviors among adolescents. The YRBS utilizes a three-stage cluster sampling method to collect a nationally representative sample of U.S. high school students from a total of 136 schools. The YRBS utilizes active and passive parental written consent and was approved by the CDC’s Institutional Review Board [26]. No additional ethics approval was needed given the data are publicly available and anonymous.

## Measures

### Dependent variable

Vaping, cigarette use, and cannabis use within the past 30 days were assessed using the combination of three items. Vaping was assessed using the question, “During the past 30 days, on how many days did you use an electronic vapor product?” Cigarette use was assessed using the question, “During the past 30 days, on how many days did you smoke cigarettes?” Cannabis use was assessed using the question, “During the past 30 days, how many times did you use marijuana?” Responses for all three items were first dichotomized to no (0) days and any (≥1) days, and then combined to create a new four-category variable that collapsed the groups to “no use,” “any single use (vaping only, cigarette use only, or cannabis use only)," “any dual use (vaping and cigarette use, vaping and cannabis use, or cigarette and cannabis use),” and “triple use (vaping, cigarette use, and cannabis use)” [2,7].

### Independent variable

Muscle-building exercise was assessed using the question, “During the past 7 days, on how many days did you do exercises to strengthen or tone your muscles, such as push-ups, sit-ups, or weight lifting?” Responses ranged from 0 to 7 days. Categories were created to capture multiple levels of engagement and to align with recommended physical activity guidance [27]. Categories included, no engagement (0 days), low engagement (1–2 days), moderate engagement (3–5 days), and high engagement (6–7 days).

#### Sociodemographic variables

Participants self-reported their age, race/ethnicity, sexual orientation, current grades, and any team sports participation in the past 12 months. Body mass index (BMI) percentile was calculated (kg/m^2^) based on self-reported height and weight. These variables were controlled for due to theoretical and empirical relevance to the independent and dependent variables. All analyses were stratified by sex given differing prevalence of muscle-building exercise and substance use behaviors among adolescent boys and girls [8,12,15,16,28–31]. Additionally, we tested for effect modification by sex and found significant associations further supporting stratifying the analyses.

### Statistical analysis

Descriptive statistics were conducted among the overall sample, and by sex, with differences between male and female participants determined using the adjusted *F*, a variant of the second-order Rao-Scott adjusted χ^2^ statistic. A total of three multinomial logistic regression analyses were conducted (one among the overall sample and two stratified by sex). Relative risk ratios (RRR) and corresponding 95% confidence intervals (CI) were used to determine the adjusted associations between muscle-building exercise and the four-category concurrent use variable among participants, while adjusting for the sociodemographic variables. The four-category concurrent use variable was used in regression analyses given small cell sizes in the expanded substance use variable. To account for multiple comparisons, we used the Benjamini-Hochberg procedure with a false-discovery rate of 10% [32]. We tested for differences between those included and those missing (i.e., those who did and did not complete the muscle-building exercise item) and the four-category concurrent substance use variable, by sex, and found no significant differences (all p > 0.05). For the remaining missing values, listwise deletion was used, which is robust to the missing at random assumptions and raises minimal issues with statistical power given the sample size of the study [33,34]. Missing values for each variable are shown in S1 Table. All analyses included preconstructed nationally representative sample weighting, including both cluster and strata variables as recommended by the CDC [26], and were conducted in 2022 using Stata 17.

## Results

Overall, the sample was comprised of 50.3% male, 52.9% White, non-Hispanic, 25.4% 16 years old, and 84.9% heterosexual. Sample characteristics among the overall sample, and stratified by sex, are displayed in Table 1. Males reported significantly higher prevalence of both moderate engagement (3–5 days; 35.5%) and high engagement (6–7 days; 23.5%) in muscle-building exercise compared females (28.9% and 10.8%, respectively). A majority of both males (63.0%) and females (61.3%) reported no vaping, cigarette use, nor cannabis use; however, males (4.4%) reported higher prevalence of triple use compared to females (3.2%).

**Table 1:** Characteristics of U.S. high school students from the 2019 Youth Risk Behavior Survey (N = 8,474).

	Overall	Male	Female	p
	%	%	%	
Sex				
Male	50.3	-	-	
Female	49.7	-	-	
Age				0.074
≤ 14 years	12.1	11.3	12.8	
15 years	24.8	25.2	24.3	
16 years	25.4	25.6	25.1	
17 years	23.8	23.0	24.7	
≥ 18 years	13.9	14.9	12.9	
Race/ethnicity				0.018
White, non-Hispanic	52.9	53.4	52.4	
Black or African American	9.2	10.1	8.3	
Hispanic/Latino/a^a^	27.9	26.8	29.1	
Asian	4.8	4.8	4.8	
Other^b^	0.9	1.1	0.7	
Non-Hispanic multi-racial	4.2	3.7	4.7	
Sexual orientation				< 0.001
Heterosexual	84.9	91.6	78.3	
Gay or lesbian	2.2	1.8	2.5	
Bisexual	8.8	3.6	13.9	
Not sure	4.1	3.0	52	
BMI percentile				< 0.001
Underweight (BMI < 5th percentile)	2.5	3.2	1.9	
Normal weight (5th ≤ BMI < 85th percentile)	58.1	56.0	61.2	
Overweight (85th ≤ BMI < 95th percentile)	14.2	12.9	15.7	
Obese (BMI > 95th percentile)	25.2	27.9	21.2	
Current grades				< 0.001
Mostly A’s	38.7	33.3	44.3	
Mostly B’s	37.1	38.7	35.5	
Mostly C’s	16.1	18.5	13.5	
Mostly D’s	3.4	4.5	2.3	
Mostly F’s	1.3	1.6	0.9	
Not sure/none of these	3.4	3.4	3.4	
Any team sports participation, past 12 months	57.9	60.9	54.8	< 0.001
Days of muscle-building exercise, past 7 days				< 0.001
No engagement (0 days)	29.7	23.7	35.8	
Low engagement (1–2 days)	20.8	17.4	24.4	
Moderate engagement (3–5 days)	32.3	35.5	28.9	
High engagement (6–7 days)	17.2	23.5	10.8	
Concurrent substance use, past 30 days				0.001
No use	62.1	63.0	61.3	
Any single use	19.4	17.4	21.3	
Vaping only	14.9	12.7	17.1	
Cigarette use only	0.4	0.5	0.3	
Cannabis use only	4.1	4.2	4.0	
Any dual use	14.7	15.2	14.2	
Vaping and cigarette use	1.4	1.6.	1.2	
Vaping and cannabis use	13.1	13.3	12.8	
Cigarette and cannabis use	0.2	0.3	0.2	
Triple use	3.8	4.4	3.2	

Note: Analyses included preconstructed nationally representative sample weighting.

BMI = Body mass index.

Differences between sexes were determined using the adjusted *F*, a variant of the second-order Rao-Scott adjusted χ^2^ statistic.

^a^Hispanic/Latino/a included multi-racial Hispanic/Latino/a.

^b^Other race/ethnicity included American Indian, Alaskan Native, Native Hawaiian, or other Pacific Islander.

Results from adjusted analyses revealed significant associations between muscle-building exercise and concurrent substance use among participants. Among the overall sample (Table 2), compared to no engagement in muscle-building exercise, participants who reported moderate engagement (3–5 days; RRR 1.42, 95% CI 1.16–1.75) and high engagement (6–7 days; RRR 1.36, 95% CI 1.06–1.72) had greater relative risk of single use versus no use. Regarding dual use, compared to no engagement in muscle-building exercise, participants who reported moderate engagement (3–5 days; RRR 1.35, 95% CI 1.07–1.71) and high engagement (6–7 days; RRR 1.46, 95% CI 1.10–2.94) had greater relative risk of dual use versus no use. Lastly, regarding triple use, compared to no engagement in muscle-building exercise, participants who reported high engagement (6–7 days; RRR 1.81, 95% CI 1.05–3.12) had greater relative risk of triple use versus no use.

**Table 2:** Associations between muscle-building exercise and concurrent substance use among participants from the 2019 Youth Risk Behavior Survey (N = 8,474).

	No use vs. Single use		No use vs. Dual use		No use vs. Triple use	
	RRR (95% CI)	p	RRR (95% CI)	p	RRR (95% CI)	p
Days of muscle-building exercise, past 7 days						
No engagement (0 days)	Ref.	Ref.	Ref.	Ref.	Ref.	Ref.
Low engagement (1–2 days)	1.06 (0.84–1.33)	0.647	1.27 (0.99–1.62)	0.062	1.14 (0.70–1.86)	0.590
Moderate engagement (3–5 days)	**1.42 (1.16–1.75)**	**0.001**	**1.35 (1.07–1.71)**	**0.013**	1.10 (0.67–1.80)	0.698
High engagement (6–7 days)	**1.36 (1.06–1.72)**	**0.017**	**1.46 (1.10–2.94)**	**0.009**	**1.81 (1.05–3.12)**	**0.033**

Note: Analysis included preconstructed nationally representative sample weighting. **Boldface** indicates statistical significance based on Benjamini-Hochberg procedure.

No use = No vaping, cigarette use, nor cannabis use.

Single use = Vaping only, cigarette use only, or cannabis use only.

Dual use = Vaping and cigarette use, vaping and cannabis use, or cigarette and cannabis use.

Triple use = Vaping, cigarette use, and cannabis use.

RRR = Relative risk ratio; CI = Confidence interval.

Analysis adjusted for age, sex, race/ethnicity, sexual orientation, body mass index percentile, current grades, and any team sports participation.

In adjusted analyses stratified by sex (Table 3), associations between muscle-building exercise and concurrent substance use were primarily among males. Regarding single use, compared to no engagement in muscle-building exercise, males who reported moderate engagement (3–5 days; RRR 1.63, 95% CI 1.17–2.25) and high engagement in muscle-building exercise (6–7 days; RRR 1.76, 95% CI 1.21–2.57) had greater relative risk of single use versus no use. Regarding dual use, males who reported moderate engagement (3–5 days; RRR 1.46, 95% CI 1.02–2.07) and high engagement in muscle-building exercise (6–7 days; RRR 1.69, 95% CI 1.14–2.51) had greater relative risk of dual use versus no use. Finally, regarding triple use, males who reported high engagement in muscle-building exercise (6–7 days; RRR 2.19, 95% CI 1.05–4.57) had greater relative risk of triple use versus no use.

**Table 3:** Associations between muscle-building exercise and concurrent substance use among male and female participants from the 2019 Youth Risk Behavior Survey (N = 8,474).

	No use vs. Single use				No use vs. Dual use				No use vs. Triple use			
	Male		Female		Male		Female		Male		Female	
	RRR (95% CI)	p	RRR (95% CI)	p	RRR (95% CI)	p	RRR (95% CI)	p	RRR (95% CI)	p	RRR (95% CI)	p
Days of muscle-building exercise, past 7 days												
No engagement (0 days)	Ref.	Ref.	Ref.	Ref.	Ref.	Ref.	Ref.	Ref.	Ref.	Ref.	Ref.	Ref.
Low engagement (1–2 days)	1.14 (0.77–1.72)	0.504	1.02 (0.76–1.35)	0.908	1.51 (1.00–2.27)	0.050	1.16 (0.85–1.59)	0.355	1.54 (0.77–3.06)	0.255	0.88 (0.44–1.79)	0.772
Moderate engagement (3–5 days)	**1.63 (1.17–2.25)**	**0.004**	1.33 (1.01–1.75)	0.043	**1.46 (1.02–2.07)**	**0.037**	1.33 (0.96–1.85)	0.090	1.05 (0.52–2.11)	0.923	1.29 (0.65–2.56)	0.461
High engagement (6–7 days)	**1.76 (1.21–2.57)**	**0.003**	0.99 (0.68–1.43)	0.961	**1.69 (1.14–2.51)**	**0.010**	1.17 (0.75–1.83)	0.483	**2.19 (1.05–4.57)**	**0.037**	1.15 (0.46–2.88)	0.762

Note: Analysis included preconstructed nationally representative sample weighting. **Boldface** indicates statistical significance based on Benjamini-Hochberg procedure.

No use = No vaping, cigarette use, nor cannabis use.

Single use = Vaping only, cigarette use only, or cannabis use only.

Dual use = Vaping and cigarette use, vaping and cannabis use, or cigarette and cannabis use.

Triple use = Vaping, cigarette use, and cannabis use.

RRR = Relative risk ratio; CI = Confidence interval.

Analysis adjusted for age, race/ethnicity, sexual orientation, body mass index percentile, current grades, and any team sports participation.

## Discussion

The results from this study report varying patterns of association between muscle-building exercise and concurrent substance use behaviors among U.S. adolescents. While there were significant findings among the overall sample, stratified analyses emphasized that the relationship between muscle-building exercise and patterns of concurrent substance use were particularly driven males. Specifically, high engagement (6–7 days) in muscle-building exercise was associated with any single use, any dual use, and over two-fold greater relative risk of triple use, compared to no use. Similarly, moderate engagement (3–5 days) in muscle-building exercise was associated with any single use and any dual use, compared to no use. Taken together, these findings contrast with prior research in the U.S., which has shown that male and female dual users of e-cigarettes and cigarettes have a lower likelihood of muscle-building exercise [8]. However, these findings align with research in Canada showing associations between muscle-building exercise and vaping among males [35].

The findings from this study underscore that muscle-building exercise, unlike other forms of physical activity [8] and sports participation [11], may not be protective of substance use behaviors, including vaping, cigarette use, and cannabis use, particularly among males. In fact, muscle-building exercise was associated with a higher risk of any single use and any dual use among males even at moderate participation rates, which generally align with recommended guidance from the American Academy of Pediatrics (i.e., 2–3 non-consecutive days per week) [27]. However, it is concerning that males who reported high engagement in muscle-building exercise, that is engaged 6–7 days per week, which is thus outside the recommended guidance, had greater risk of all substance use patterns, underscoring a potentially problematic clustering of behaviors as outlined by problem behavior theory [20,21]. These findings add the list of potential risk factors of substance use behaviors among adolescents [2].

Prior research has shown that adolescent use of vaping and cigarettes are mechanisms for appetite control or weight loss, and connected with weight concerns [36,37]. Thus, given the sociocultural prominence of the muscular and lean body ideal among males [16,38], vaping, cigarette use, and cannabis use may be adjunct behaviors to muscle-building exercise intended to achieve this body ideal. Additionally, adolescent males may use muscle-building exercise and substance use behaviors to display their adherence to masculine gender norms, such as strength, toughness, and risk-taking behavior [22]. Indeed, future research is needed to explicate the mechanisms (i.e., sensation seeking, peer influence, attempts to adhere to masculine norms) underlying the associations found in this study, as well as whether these substance use behaviors are associated with other forms of muscularity-oriented behaviors (e.g., protein overconsumption, muscle-building supplement and substance use). Indeed, muscle-building exercise may also be a proxy for other behaviors (i.e., energy drink use), which have been shown to be associated with vaping, cigarette use, and cannabis use [39,40].

Strengths of this study include a large, nationally representative sample of U.S. adolescents. Despite these strengths, several limitations should be noted. The YRBS data are cross-sectional, which limits directional associative inference. Additionally, there were differing time frames assessed by the vaping, cigarette use, and cannabis use measures, as well as the muscle-building exercise measure (i.e., within the past 30 days versus within the past 7 days). Thus, there may be gaps between the substance use and engagement in this form of exercise, leading to an attenuation in the current associations. Future research should utilize a prospective cohort study to assess and identify how the associations occur and evolve over time. All items were based on retrospective self-report, which may increase the risk of reporting and recall bias. Additionally, given small cell sizes, we were unable to investigate whether muscle-building exercise was associated with individual, mutually exclusive substance use categories (i.e., vaping only, cigarette use only, etc.) in adjusted analyses, which is a line of inquiry for future research. The YRBS is conducted only in the United States, which limits external validity to other populations (i.e., countries). Lastly, there is the potential for unmeasured confounders, such as sensation seeking and peer influence, that may have influenced the findings.

Considering these limitations, the findings from this study have important implications. Adolescents, particularly males, should seek professional instruction and oversight when participating in muscle-building exercise to ensure proper and safe engagement, potentially reducing other problematic behaviors (i.e., concurrent substance use). Education and training for professionals, including coaches and physical trainers, are needed to bring awareness of the potential risks of concurrent substance use associated with muscle-building exercise. Coaches and physical trainers may also require training on proper intervention strategies, such as assessment and referral processes, to aid adolescents who participate in moderate and high levels of muscle-building exercise and are susceptible to concurrent substance use. Despite potential benefits of muscle-building exercise, healthcare providers should be aware of the unique relationship between this form of exercise and substance use behaviors when treating adolescent males, particularly when engagement in muscle-building exercise is moderate or high. Public health professionals should consider including information on concurrent substance use into physical activity education for adolescents.

## Conclusion

This study aimed to determine whether muscle-building exercise is associated with patterns of concurrent use of vaping, cigarette use, and cannabis use among a nationally representative sample of U.S. adolescents. Findings showed that muscle-building exercise was associated with varying patterns of concurrent use, particularly among adolescent males. Of particular concern is the strong relationship between high engagement in muscle-building exercise and triple use of e-cigarettes, cigarettes, and cannabis. Findings underscore the need for more research and prevention and intervention efforts to address this potentially problematic relationship.

## Supporting information

S1 Table Missing values by variable. (DOCX)
