# Supplementary material for: Associations between muscle-building exercise and concurrent e-cigarette, cigarette, and cannabis use among U.S. adolescents
Source: PLoS One. 2022 Dec 28;17(12):e0278903. doi: 10.1371/journal.pone.0278903 (PMC9797070; doi:10.1371/journal.pone.0278903)
Supplement: S1 Table — (DOCX) [file pone.0278903.s001.docx]

| S1 Table  Missing values by variable | | |
| --- | --- | --- |
| Variable name | Number missing/Total observations | % missing |
| Sex | 66/8,474 | 0.8 |
| Age | 49/8,474 | 0.6 |
| Race/ethnicity | 215/8,474 | 2.5 |
| Sexual orientation | 491/8,474 | 5.8 |
| BMI percentile | 0/8,474 | 0.0 |
| Current grades | 224/8,474 | 2.6 |
| Team sports participation, past 12 months | 59/8,474 | 0.7 |
| Concurrent substance use, past 30 days | 562/8,474 | 6.6 |
